# An Algorithmic Approach for Quantitative Determination of Microsatellite Status in NGS-Based Cancer Diagnostics

**DOI:** 10.3390/cancers18030433

**Published:** 2026-01-29

**Authors:** Josefin Männlein, William Sterlacci

**Affiliations:** Institute of Pathology, Medizin Campus Oberfranken, Klinikum Bayreuth, Friedrich-Alexander-Universität Erlangen-Nürnberg (FAU), 95445 Bayreuth, Germany

**Keywords:** microsatellite instability, targeted NGS, mismatch repair, MSI detection, bioinformatics, colorectal cancer

## Abstract

Purpose: To develop a transparent and adaptable methodological framework for the analysis of microsatellite status using Next Generation Sequencing (NGS), addressing current limitations in clinical implementation. Methods: Microsatellite status was assessed using NGS with a custom-designed panel. The approach was validated against polymerase chain reaction (PCR) and immunohistochemistry (IHC) results in a cohort of 32 patients with various tumor entities. A Python-based analysis pipeline was developed to process raw sequencing data and quantify mutational burden within microsatellite regions. Results: The proposed method demonstrated 100% concordance with PCR and 90.32% concordance with IHC results. The framework enabled quantitative assessment of microsatellite instability. Conclusions: This proof-of-concept study demonstrates reliable determination of microsatellite status. The transparent and panel-adaptable framework offers flexibility for clinical implementation and provides a robust foundation for further validation in larger cohorts and across diverse tumor entities.

## Simple Summary

Determining microsatellite instability is an essential component of modern cancer diagnostics, particularly for identifying patients who may benefit from immunotherapy. While Next Generation Sequencing offers the possibility to assess microsatellite status alongside other molecular markers, its clinical use is often limited. In this study, we present a transparent and adaptable framework for microsatellite analysis. The approach was validated against established diagnostic methods and showed high agreement. Beyond binary classification, it enables a quantitative assessment of microsatellite alterations and flexible threshold definition. This framework may facilitate the integration of sequencing-based analysis into routine diagnostics.

## 1. Introduction

In oncological diagnostics, the determination of microsatellite status has become a key component, serving both as a predictive and prognostic biomarker. Microsatellite instability (MSI) arises when the DNA mismatch repair (MMR) system is compromised, leading to an accumulation of mutations within repetitive DNA sequences known as microsatellites [1,2].

The resulting high mutational burden within these regions enhances the visibility of tumor cells to the immune system [3]. This, in turn, often triggers the development of immune evasion strategies, notably through upregulation of the PD-1/PD-L1 checkpoint pathway [4]. The therapeutic blockade of this pathway using immune checkpoint inhibitors, such as pembrolizumab or nivolumab, has consequently been associated with improved clinical outcomes in MSI tumors of various entities [1].

As a result of these findings, the clinical demand for analysis of microsatellite status continues to increase, leading international guidelines like the 2025 German S3 guideline to recommend universal MSI testing for all colorectal carcinomas [5]. Moreover, the expanding approvals of checkpoint inhibitors for multiple tumor entities underscore the need for more targeted diagnostic strategies [6,7].

Current standard methods for MSI determination include immunohistochemistry (IHC) for evaluation of MMR protein expression, as well as polymerase chain reaction (PCR), which detects alterations in specific microsatellite loci using established marker panels such as Bethesda or Pentaplex [8,9].

In addition to these established approaches, Next Generation Sequencing (NGS) is increasingly applied, as it offers a more comprehensive molecular profiling by being able to analyze multiple diagnostic genes [10,11]. For the evaluation of raw data generated using NGS several open-source tools, including MANTIS [12], mSINGS [11], as well as MSIsensor [10] have been developed. Although all of the tools mentioned are used in research and clinical settings, substantial gaps persist between technological capability and clinical implementation.

Despite the increased availability of MSI detection tools in recent years, NGS is regarded primarily as supplementary in diagnostics regarding determination of microsatellite status [13]. Recent systematic evaluations have begun to characterize specific limitations of established tools such as specific cutoffs, panel-dependent adjustment and the need for more adaptable strategies [14].

Furthermore, the optimal selection of microsatellite loci differs between various tumor types, and established tools may require a larger number of loci for valid analysis than present in common clinical panels [10].

Beyond technical performance, barriers to broader NGS adoption also include bioinformatic complexity in data interpretation [15], cost considerations, as well as limited transparency of algorithmic decision-making [16].

In cases with discordant results or atypical patterns of MMR protein loss several studies suggest that incorporating NGS in the diagnostic process could be valuable [7]. The broader and more flexible selection of genes, together with the potential integration of additional markers such as tumor mutational burden (TMB), could provide complementary diagnostic insights and therefore enable the interpretation of ambiguous cases [17]. However, regarding the quantification of microsatellite status rather than consideration as a dichotomous variable, no definitive consensus on clinical significance has yet been reached [18].

In this context, the present study aimed to develope a transparent, panel-adaptable framework for NGS-based MSI determination that performs on panel sizes representative of routine clinical practice. The focus of this work lies in enhancing methodological flexibility and enabling the potential integration of additional biomarkers.

## 2. Materials and Methods

The study was conducted in accordance with the ethical principles outlined by the Ethics Committee of Friedrich-Alexander University Erlangen-Nürnberg (approval number 23-75-Br, dated 21 March 2023).

Patient data were pseudonymized to ensure confidentiality, and all analyses were performed strictly for research purposes. For molecular analysis, a cohort of 32 patients who had previously undergone routine MSI testing, was selected. The cohort included both microsatellite-stable (MSS) as well as -instable cases (Table 1), encompassing typical and atypical immunohistochemical expression patterns (Table 2).

Patients with various tumor entities were represented in the cohort (Table 3). Retrospective clinical data, including IHC- and PCR test results, were collected for all patients. One patient was subsequently excluded from the final analysis of microsatellite status due to insufficient tumor cell content in the specimen.

Of the 32 included patients, 28 (87.5%) were diagnosed with colorectal adenocarcinomas, while only four patients represented other tumor entities. This imbalance in tumor types was deemed acceptable for the purposes of initial methodological validation. However, it necessarily constrains the generalizability of performance metrics and the applicability of established cutoff values to broader clinical populations.

The present proof of concept was designed to validate a transparent framework, as well as demonstrating methodological feasibility, while acknowledging that full clinical implementation would require further validation in larger and independent patient cohorts.

Concordance rates, sensitivity and specificity of the NGS-based approach were statistically assessed against IHC and PCR results to evaluate the diagnostic validity.

Microsatellite status was assessed using PCR-based fragment length analysis. The classical Bethesda panel markers BAT-25, BAT-26, D17S250, D2S123, and D5S346 were analyzed. In addition, a Pentaplex panel comprising NR-21, NR-24, and NR-27 was used. In selected cases, the markers CAT-25 and NR-22 were included.

MSI-high (MSI-H) was defined as instability in ≥30% of analyzed markers, corresponding to at least two unstable markers in a five-marker panel. Tumors with instability below this threshold were classified as MSS, as MSI-low (MSI-L) cases were grouped with MSS.

An overview of all PCR markers is provided in Table 4.

### 2.1. Panel Composition

NGS was performed using a custom panel (MSI Cancer HotSpot Panel v2, Illumina, San Diego, CA, USA), which comprised 130 microsatellite loci distributed across diagnostically relevant cancer-associated genes. The complete panel composition is provided in Supplementary Materials Table S1.

The panel consisted of two primer pools at a concentration of 2×400 µM covering a total of 133 amplicons.

This number of microsatellite loci was examined to achieve a broad and informative assessment of MSI and its potential association with cancer-related genes. Nevertheless the approach remains adaptable and permits future reduction and validation of smaller panels.

The custom panel was further modified with the aim to assess whether MSI can be reliably determined by analysis of microsatellite loci within cancer-associated genes, rather than relying on classical Bethesda or Pentaplex markers or inclusion of MMR genes.

Mononucleotide repeats of sufficient length, typically exceeding a number of ten repeat units, were preferred, which have been shown to provide higher sensitivity than dinucleotide repeats [8,9]. Additionally, the *HSP110* gene was included in the panel due to monomorphic structure, which is able to achieve up to 98.4% sensitivity and 99.7% specificity for MSI detection [19].

### 2.2. DNA Isolation and Sequencing

To generate the NGS library, DNA was amplified in a multiplex PCR using DNA polymerase. The amplification products were purified using 30 µL AMPure XP Beads. Samples were indexed in a subsequent PCR for multiple cycles using the appropriate primers. After final purification with 30 µL AMPure XP Beads, the resulting PCR products constituted a sequencing-ready library compatible with the selected Illumina platform.

Analysis of the NGS run revealed a cluster density of 375 clusters/mm^2^, of which 95.3% passed the filter. Additionally, 91.8% of bases met the quality threshold.

A Python-based evaluation pipeline was developed for the analysis of NGS-generated data. Raw sequencing data were aligned to a reference genome, and microsatellite status was determined by quantitatively assessing the mutational burden within microsatellite regions, including insertions, deletions, and base substitutions.

Following sequencing, raw NGS data were obtained as FASTQ files. To facilitate downstream analyses, FASTQ files were converted into standardized formats, either SAM (Sequence Alignment/Map) or its binary counterpart, BAM (Binary Alignment/Map).

For further processing and evaluation of these files, additional bioinformatics tools were employed. In this study, SAMtools was utilized for the operations, as described in detail by [20].

The Python (v3.12) script used to evaluate the microsatellite status is available on GitHub: https://github.com/josefinmn25/NGS_Analysis/tree/main (accessed on 30 November 2025).

### 2.3. Preprocessing and Filtering of Reads

Raw NGS data derived from sequenced tumor DNA were converted into BAM/SAM format at the beginning of the analysis. These files contained the aligned sequence information obtained after mapping to the reference genome using the Illumina platform.

Data processing was performed using the Python library “pysam”.

The initial preprocessing step involved converting the original BAM file into SAM format to enable text-based inspection of the sequencing information. Data were processed line by line, with each line representing a sequenced read in SAM format. Each read comprises multiple SAM fields that convey specific information, the most relevant of which include:

- FLAG: Encodes alignment properties, read pairing status, and mapping characteristics
- MAPQ: Indicates the mapping quality of the read
- CIGAR: Describes the alignment of the read to the reference genome, including matches, insertions, deletions, and soft or hard clipping
- MD:Z: Represents mismatches between the read and the reference sequence, useful for identifying single-nucleotide variants
- NM:i: Specifies the total number of deviations from the reference genome, including mismatches and insertions as well as deletions

Several criteria were applied to filter the generated reads. A key parameter was the mapping quality (MAPQ), which reflected the probability that a read has been correctly aligned to the reference genome [21]. In this study, only reads with a MAPQ score ≥60 were retained to ensure high reliability and minimize the risk of misalignment that could lead to misinterpretation.

The second filtering criterion involved the use of bit flags, which encode multiple attributes of each read in binary form, allowing precise determination of the read’s status. For valid analysis, only reads meeting the following criteria were considered:

- Primary alignment (FLAG: 0 × 100): Only primary alignments are retained; secondary alignments representing alternative mapping positions are excluded
- Non-supplementary (FLAG: 0 × 800): Supplementary alignments, such as fragments from split reads, are excluded from the analysis
- Mapped (FLAG: 0 × 4): Only mapped reads are considered, as unmapped reads lack usable positional information

The binary-encoded bit flags were converted into decimal values, with each value assigned its corresponding read-specific information using a standard binary-to-decimal converter.

### 2.4. Counting Valid Reads

The subsequent step involved determining the total number of reads available for each microsatellite locus to enable quantitative mutation analysis. This was achieved by filtering reads based on chromosomal location and precise genomic coordinates of the respective genes, as determined by the predefined panel.

### 2.5. Determination of the Reference Sequence

In the next step, reads matching the corresponding region of the reference genome (reads without mutations or sequence alterations) were identified. For each gene, the presence of such a read among the sequenced data was assessed to determine whether it could serve as a reference. This was determined by examining the MD tag described above. Reads with an MD tag indicating no mismatches were classified as reference reads.

MD tags without mismatches signified that all bases in the read aligned perfectly with the reference genome. Alternatively, the CIGAR string could provide similar information. If the CIGAR string only contained match operations, the read was considered identical to the reference genome [22].

Because BAM/SAM files typically only document differences relative to the reference genome and do not include the original reference, reconstruction of the latter was necessary, if no read without mutations could be identified. This reconstructed sequence served as the basis for identifying microsatellite regions and quantifying mutations within them.

Reconstruction was performed by analyzing the read-to-reference relationship using both the CIGAR string and the MD tag. As described above, the CIGAR string specified how the read bases aligned with the reference genome and provided information on genomic alterations, including M (matches), I (insertions), D (deletions), and S (soft-clipped bases at read ends).

Soft-clipped bases were removed in a subsequent step due to their irrelevance for microsatellite analysis. The MD tag provided information on base modifications and deletions relative to reference coordinates [22].

### 2.6. Quantification of Microsatellite Instability

To quantitatively assess potential microsatellite instability, ratios representing the mutational burden within the microsatellite region were calculated. The analysis was conducted in two dimensions: first, considering the number of mutated reads per locus, and second, evaluating all microsatellite mutations relative to the total number of reads. For each dimension, the mean percentage across all genes included in the panel was determined. Based on these mean values across all patients, a cutoff value was established for each metric using receiver operating characteristic (ROC) analysis, above which patients were classified as MSI or MSS.

**Figure 1:**
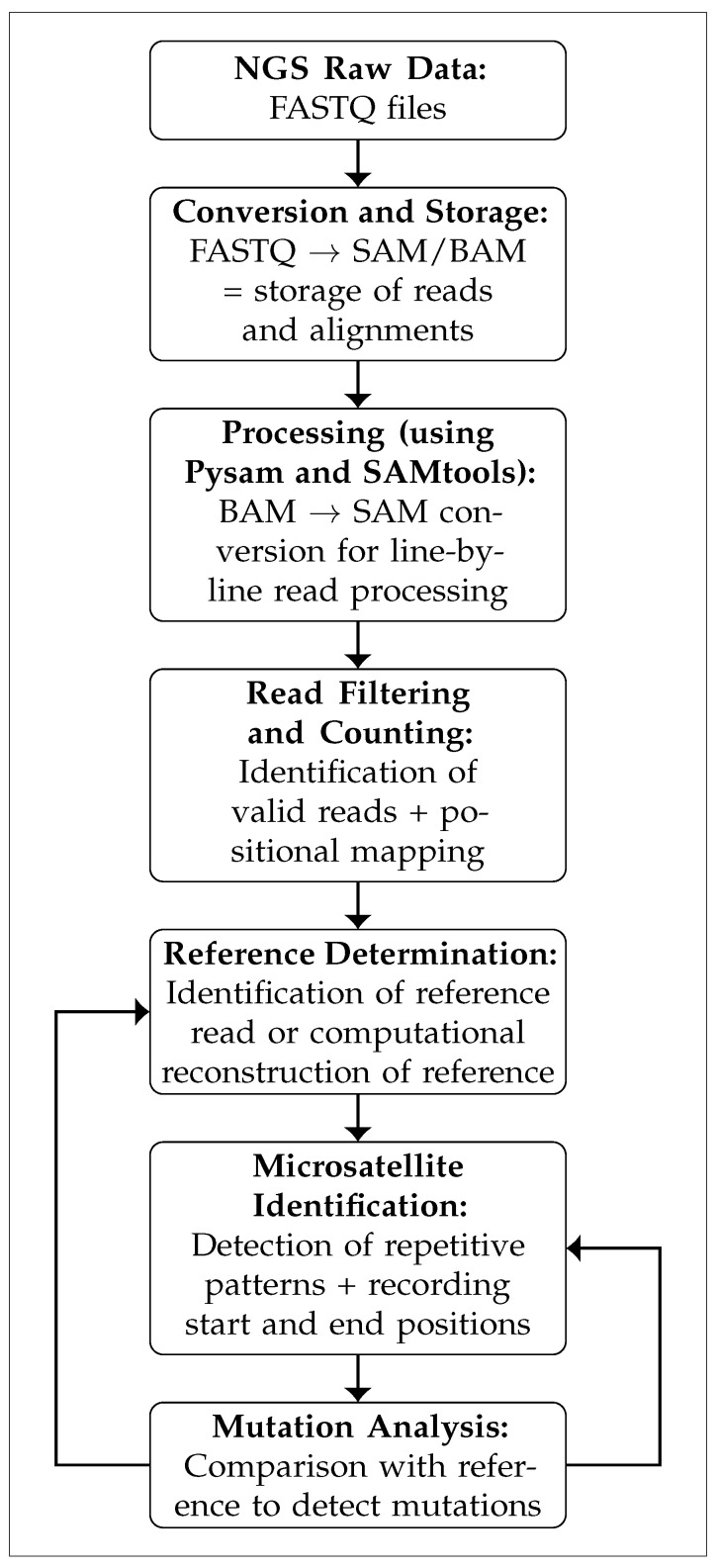
Workflow for NGS-based microsatellite analysis.

The described analysis process is illustrated in Figure 1.

This approach was validated by comparing the results with established PCR and IHC methods from the same samples. Statistical analyses were conducted using SPSS (v29.0.1.0), and data visualization was performed using LaTeX (MiKTeX 25.4).

#### Functional Testing

To ensure the functional correctness of the script, a series of self-designed test cases were implemented. These test cases were designed to simulate defined sequencing scenarios that could lead to possible errors in the evaluation and contained insertions, deletions, and base substitutions in and around microsatellite regions. Their purpose was to verify the script’s handling of positional shifts, CIGAR string complexity, and MD tag interpretation.

Three representative test scenarios were created:

- Test Case 1: Evaluated whether insertions or deletions upstream of the microsatellite region affected the correct identification of mutations within the repeat region
- Test Case 2: Assessed the ability to detect and correctly count multiple independent insertions or deletions within a microsatellite tract
- Test Case 3: Investigated whether indels located at the beginning of a microsatellite were interpreted as part of the microsatellite sequence

For each of these, both the respective reference and read sequences were defined, including the simulated mutations, CIGAR strings, and MD tags. The expected outputs were compared with the actual script output, serving as a validation benchmark.

## 3. Results

An independent evaluation method for determining microsatellite status was developed based on the analysis of raw sequencing data in SAM/BAM format.

The method employed Python-based analysis, without reliance on external software. The flexible panel design enabled targeted selection of diagnostically relevant genes for further information.

### 3.1. Performance of NGS-Based Analysis

Validation was conducted using 32 FFPE tumor samples from various tumor entities. Results obtained with the NGS-based method were compared to those of standard PCR and IHC assays. Performance metrics included sensitivity, specificity, as well as concordance rates. NGS analysis was successfully completed for 31 samples. Regarding the developed evaluation method, complete agreement was achieved with PCR results (100%), while concordance with IHC results was 90.32%. Sensitivity and specificity were 100% for PCR comparison and 100% and 82.35% for IHC comparison, respectively (Table 5).

With regard to the final classification of microsatellite status, 100% agreement was found between PCR-based and NGS-based analyses. While PCR defined MSI as instability in ≥30% of the markers used, NGS referred to the self-calculated thresholds, which depended on the loci used. PCR-based testing identified instability within classical Bethesda and Pentaplex markers (Table 4), whereas NGS detected instability across a broader set of microsatellite loci located in cancer-associated genes. Therefore, the classification of microsatellite status was based on different loci and corresponding cutoffs, accordingly the resulting concordance was based only on the final classification as MSI or MSS.

Overall concordance between IHC and molecular MSI testing was 90.32%. Three cases showed discordant results between IHC and molecular analyses, whereby these tumors were classified as MSI by both molecular methods but showed proficient MMR by IHC.

The method further enabled quantification of the mutational burden within microsatellite regions, expressed as the proportion of the total amount of mutations within the reads relative to total reads. Further information on the evaluation of the raw data can be found in Supplementary Materials Table S2.

### 3.2. Most Altered Genes

The ten most altered genes within the used panel are shown in Table 6.

### 3.3. Determination of Cutoff Value

To determine whether a sample was to classify as MSS or MSI, the ratio of mutated reads to total reads, as well as the ratio of total mutations within the microsatellite regions to total reads, was calculated for each gene. Subsequently, the mean of these ratios across all genes was computed for each patient, and a cutoff value was established to enable comparability between NGS and PCR results. For the NGS data, threshold analysis was conducted to identify the optimal percentage at which a patient would be classified as MSI. This was determined using a ROC curve and the corresponding cutoff derived therefrom.

ROC curves were generated separately for the total number of mutations within microsatellite regions and for the number of mutated reads.

The ROC analysis assessing the diagnostic performance of the molecular test (NGS: ratio of mutated reads to total reads, in %) yielded an area under the curve (AUC) of 0.967. The optimal cut-off value for distinguishing MSI from MSS cases based on the maximum Youden index and was 44.72% (Youden index: 0.933).

When considering the total number of mutations relative to the total number of reads, ROC analysis for the parameter “ratio of total mutations within microsatellite regions to total reads, in %” yielded an AUC of 0.954. The optimal threshold, likewise determined using the maximum Youden index, was 71.00% (Youden index: 0.933).

The outcomes of the NGS analysis were dichotomized whereby patients below the cut-off were classified as MSS, and patients above the cutoff as MSI.

More detailed information on individual cutoff values of the used genes can be found in Supplementary Materials Table S3.

### 3.4. Results of Test Cases

The self-developed test cases confirmed that the algorithm correctly identified mutations across all simulated situations. The details of the test cases can be found in the Appendix A.

## 4. Discussion

With the expanding therapeutic use of immune checkpoint inhibitors in tumors exhibiting MSI, the demand for accurate and flexible diagnostics is steadily increasing. Although PCR and IHC remain established standard methods, NGS is gaining relevance as a complementary molecular diagnostic approach that enables characterization of microsatellite status while gaining additional genetic information.

This study presents a proof-of-concept evaluation of a transparent, panel-adaptable framework for NGS-based MSI diagnostics. While showing methodological feasibility, the need for validation in substantially larger, independent cohorts to establish robust performance as well as the standardization of technical parameters (e.g., sequencing depth requirements, reporting standards) should be addressed before clinical implementation.

The panel was not restricted to classical MMR genes, but instead targeted multiple microsatellite regions. In addition to offering cost efficiency, particularly in resource-limited settings, this approach allows for modification to address different clinical questions.

The detailed presentation of the evaluation workflow and validation data enhances the transparency and reproducibility of the diagnostic methodology. While external software tools for NGS data analysis, such as the open-source programs MANTIS or MSIsensor [10,12], are used in clinical practice and provide reliable assessment, their underlying algorithms often remain non-transparent and complex [15,16].

Despite the increased availability of NGS-based MSI tools in the recent years, a study by Austin et al. revealed that only 9.6% of US laboratories employ NGS as their primary MSI testing method, compared to 87.2% using IHC and 40.4% PCR [13]. This limited uptake, despite the increasingly widespread use of checkpoint inhibitors and the resulting requirement for MSI determination [7], suggests that important practical requirements remain unaddressed.

The recent study by Anthony and Seoighe evaluated widely used bioinformatic tools, including MANTIS and MSIsensor, and highlighted their limitations by showing a loss of accuracy when applied to panels differing from those used in their original training. This dependency is partly attributable to tool-specific cutoffs, which require adjustment according to the sequencing approach. The authors underscored the need for universal standards and adaptive thresholds to enable more precise and reproducible MSI determination [14].

Our approach addressed this limitation through transparent cutoff determination that can be adapted to specific panels and clinical contexts. By documenting each analytical step, the framework also facilitates analyzing of discordant cases.

Furthermore, the 130-locus panel represents an intermediate approach, bridging the gap between small conventional panels and those with more than 500 loci typically recommended for tools such as MSIsensor [10]. These algorithms optimized for large panels may experience reduced sensitivity and specificity when applied to a smaller amount of loci.

By focusing on cancer-associated genes/various microsatellite loci rather than classical MSI markers, this design also offers the potential for integration with existing oncology panels, although systematic optimization of marker selection has not yet been conducted.

Our findings demonstrate that a selected set of microsatellite loci is sufficient for accurate MSI classification. Simultaneously, targeted adaptation of loci can enhance predictive power for specific tumor types. The NGS panel used in this study comprises a variable number of microsatellite loci that can be adjusted according to diagnostic needs. Analyses revealed that certain loci exhibited higher mutation rates in specific tumor types [18]. The flexible applicability of the evaluation workflow to different panels allows genes to be tailored to distinct clinical questions and tumor entities. For instance, markers of limited relevance in a given tumor type can be excluded, while additional loci with higher sensitivity in specific subgroups can be incorporated. The MSI score is calculated based on the cumulative mutation frequency across the analyzed loci.

Hause et al. [18] identified the loci most strongly associated with MSI, including *DEFB105A/B*, *ACVR2A*, and *RNF43*. They emphasized that unstable microsatellite regions frequently occur in genes implicated in carcinogenesis, such as *ACVR2A* and *RNF43*, underscoring the relevance of including specific diagnostic genes.

At the same time, it was noted that many genes remain poorly characterized or have not been explicitly linked to MSI. Therefore, further investigation is needed, as new insights into the molecular mechanisms underlying MSI and its impact across different tumor types could be provided. Of particular interest are microsatellites located in regulatory or non-coding regions, which may influence gene expression and cellular processes through instability [18].

Subsequent work by Boyarskikh et al. pursued a similar objective with a focus on the clinical application of MSI-sensitive loci. Their study demonstrated that a small set of highly sensitive markers, including *ACVR2A*, *RNF43*, and *TGFBR2*, is sufficient to detect MSI status with high sensitivity, in some cases matching the performance of more extensive panels [23]. Together, these studies underscore the potential of locus-specific panels to enable more precise and tumor-entity-specific diagnostics.

Beyond technical performance, barriers to broader NGS adoption include bioinformatic complexity, cost considerations, and limited transparency of algorithmic decision-making [15,16]. When discordant results occur, non-transparent algorithms complicate clinical interpretation. Workflows that document analytical steps may facilitate both validation processes and resolution of ambiguous cases.

A comparable approach to MSI determination was reported by [23], who, similarly to the present study, utilized a custom Python-based script. In their work, a quantitative metric derived from microsatellite length analysis was employed to assess MSI status, and the script allowed for individualized threshold settings and tumor-specific marker optimization, providing enhanced flexibility in analysis [23].

Furthermore, the use of NGS offers considerable potential as a complementary molecular genetic approach, particularly in cases of discordant results from standard diagnostics or atypical expression losses observed in IHC [7].

Regarding the observed discordance rate (9.68%) three cases showed inconsistent results. All three cases were classified as MSI by PCR/NGS, whereas IHC showed pMMR. In one of the three cases, the results were inconclusive due to technical issues and were ultimately classified as pMMR.

These examples showing pMMR by IHC yet identified as MSI molecularly may result from epigenetic silencing of MMR genes, such as MLH1 promoter methylation, as well as technical challenges in IHC interpretation, particularly in tumors with heterogeneous staining patterns or low tumor cellularity [1,7,24,25].

NGS-based approaches offer particular value in such cases by providing quantitative assessment of microsatellite instability independent of protein expression. The ability to analyze multiple loci simultaneously and to quantify mutational burden allows for a further interpretation.

Beyond MSI assessment, additional molecular markers, such as TMB, could be incorporated into the present framework with minimal modification. High TMB is frequently observed in tumors exhibiting MSI [17]. Moreover, studies such as [26] have demonstrated that elevated TMB correlates strongly with improved response rates and prolonged progression-free survival in patients receiving PD-1/PD-L1 inhibitors, including pembrolizumab. Therefore, integrating TMB into the current NGS-based analysis could provide valuable prognostic and predictive information for therapeutic decision-making [26].

A quantitative assessment of microsatellite status allows not only classification of tumors as MSI or MSS but also differentiation of the degree of instability, potentially enabling more precise prognostic predictions and therapy stratification. However, the clinical relevance of a metric representation of microsatellite status has not yet been established, and further research is warranted.

Hause et al. [18] propose considering microsatellite status as a metric variable rather than a dichotomous phenotype, based on their observation that the mutational burden within microsatellites correlates with patient survival outcomes.

Despite these findings, the number of studies quantifying microsatellite status in a continuous manner remains limited. Most current literature continues to categorize tumors as MSI-high or MSS, occasionally including an MSI-low category, whose clinical significance remains ambiguous [9,27].

Another approach is offered by the work of [28], in which microsatellite status was not directly quantified. Instead, prognostic and therapeutic predictions were derived by correlating MSI with other quantitative data. The study focused on the immune context of the tumor, including infiltration of various immune cells and expression of immune checkpoint molecules [28].

Previous studies suggest that a high MSI degree and therefore high mutational load is generally associated with a more immunogenic tumor microenvironment, whereas a low MSI degree does not necessarily confer sensitivity to checkpoint inhibitors [29].

Other research has investigated which genes correlate most strongly with microsatellite status, enabling more differentiated insights into MSI. However, these studies have not established a direct metric representation, resulting in indirect quantitative evaluations of MSI [28].

More recently, Choi et al. [30] demonstrated that MSI can be inferred and quantified by analyzing length variations across different microsatellite regions from RNA-seq data and comparing them with normal tissue. This approach is similar to the present study, with the key distinction that the current work measures the number of mutations within microsatellite regions rather than the length variations themselves, although the two variables are inherently linked, as each mutation in a repeat sequence produces a corresponding change in length [30].

Future research into the quantitative assessment of microsatellite status holds substantial potential to improve oncological diagnostics and therapy. Both direct quantitative measurement of microsatellite instability and indirect evaluation through complementary markers, such as TMB or immunological parameters, could enable more precise prognostic assessments regarding survival or recurrence risk [31]. This enhanced prognostic power stems from the correlation between mutational burden and response to immune checkpoint inhibitors [26]. Integrating multiple molecular and immunological factors in the diagnostic workflow would support more individualized therapeutic strategies.

Furthermore, in tumors with intratumoral heterogeneity, where microsatellite status may vary across different regions [32], quantitative approaches and including additional markers could facilitate more accurate detection, ultimately improving clinical decision-making.

Taken together, these studies support the notion that microsatellite status can be considered a quantitative molecular parameter. When combined with information on immunological factors, such as tumor-infiltrating immune cells or checkpoint molecule expression, it may provide valuable insights into prognosis and therapeutic response.

Nevertheless, this study represents a methodological proof-of-concept rather than a clinically validated diagnostic tool. Although the algorithmic transparency and panel flexibility were demonstrated in principle, the relatively small cohort size and the predominance of colorectal carcinomas limit the generalizability of the performance estimates. Clinical implementation will therefore require validation in larger cohorts, inclusion of diverse tumor types, as well as entity-specific panel optimization to ensure reproducibility.

Also, the reported performance metrics and thresholds remain exploratory. Additionaly, the approach is not limited to targeted panels and could, in principle, be applied to publicly available whole-genome sequencing datasets, even if these were not originally generated for MSI assessment. Such datasets may offer valuable opportunities for large-scale benchmarking and could be addressed in future work.

The small cohort size (n = 31 evaluable cases) limits the statistical precision of performance estimates. Although the observed 100% concordance with PCR is encouraging, it should be interpreted with caution, also concerning the strong predominance of colorectal adenocarcinomas (87.5%, n = 28). The ROC-derived thresholds (44.72% for mutated reads; 71.00% for total mutations) were therefore optimized for microsatellite characteristics in this small cohort and may not translate directly to other tumor types.

Microsatellite repeat length distribution and mutation patterns vary considerably across cancers [18], which may necessitate entity-specific cutoffs.

Furthermore, genes showing high mutation frequencies in this cohort may reflect colorectal-specific patterns and require validation in other MSI-associated malignancies.

Overall, this study shows that an adaptable, transparent framework for determining MSI can be successfully applied to clinically realistic panel sizes, although full clinical implementation still needs to be validated in larger cohorts.

## 5. Conclusions

This proof-of-concept study establishes the technical feasibility of a transparent framework for NGS-based MSI determination demonstrating perfect concordance with PCR-based reference testing and high agreement with IHC.

The algorithmic approach supports reproducibility and allows adaptation to different panel compositions regarding clinical contexts. While the methodological framework is transferable, specific cutoff values remain exploratory pending validation in cohorts encompassing diverse tumor types.

In addition, the framework generates quantitative metrics of mutational burden alongside dichotomous classification, providing a foundation for future investigations into whether continuous MSI scoring may offer prognostic or predictive value beyond dichotomous categorization.

## Figures and Tables

**Table 1:** Comparison of MSI status (PCR) and MMR status (IHC) in the patient cohort.

IHC (MMR Status)	MSI (PCR)	MSS (PCR)	Total
dMMR ^1^	15	-	15
pMMR ^2^	3	14	17
Total	18	14	32

^1^ dMMR: deficient mismatch repair; ^2^ pMMR: proficient mismatch repair.

**Table 2:** MMR-protein expression patterns.

MMR Status	Expression Pattern	n
dMMR	Concurrent loss of MLH1 and PMS2	8
dMMR	Concurrent loss of MSH2 and MSH6	2
dMMR	Isolated loss of MSH6	1
dMMR	Isolated loss of PMS2	1
dMMR	Concurrent loss of MLH1, PMS2, MSH2, and MSH6	1
dMMR	Concurrent loss of MLH1, PMS2 and MSH6	1
dMMR	Concurrent loss of PMS2 and MSH6	1
Total dMMR	-	15
pMMR	-	17
Total patients	-	32

**Table 3:** Patient cohort characteristics.

Number	Year of Birth	Tumor Diagnosis	Sex	Tumor DNA Amount (ng/µL)
1	1953	ACA ^1^ Stomach	Female	69
2	1953	ACA Stomach	Female	68
3	1952	ACA Rectum	Female	71
4	1955	ACA Colon	Male	49
5	1938	ACA Colon	Female	71
6	1965	ACA Esophagus	Female	38
7	1950	ACA Colon	Female	80
8	1954	ACA Cecum	Male	92
9	1954	ACA Sigma	Female	57
10	1965	ACA Colon	Male	64
11	1937	ACA Colon	Male	77
12	1967	ACA Colon	Female	77
13	1937	ACA Cecum	Male	71
14	1936	ACA Sigma	Male	23
15	1948	ACA Rectum	Male	66
16	1967	ACA Colon	Male	310
17	1943	ACA Cecum	Female	44
18	1977	ACA Sigma/Rectum	Male	53
19	1967	ACA Sigma	Female	94
20	1952	ACA Colon	Female	33
21	1969	ACA Sigma	Male	77
22	1964	ACA Rectum	Male	65
23	1944	ACA Colon	Male	85
24	1962	ACA Rectum	Female	79
25	1978	ACA Sigma	Female	97
26	1980	ACA Sigma	Female	33
27	1943	Medullary CA ^2^ Colon	Female	91
28	1940	ACA Colon	Female	40
29	1949	ACA Colon	Male	91
30	1969	ACA Sigma	Female	33
31	1985	ACA Colon	Male	54
32	1968	ACA Cecum	Female	67

^1^ ACA: Adenocarcinoma; ^2^ CA: Carcinoma.

**Table 4:** PCR-based microsatellite markers used for MSI analysis.

Marker	Repeat Type	Panel Assignment
BAT-25	Mononucleotide	Bethesda panel
BAT-26	Mononucleotide	Bethesda panel
D2S123	Dinucleotide	Bethesda panel
D5S346	Dinucleotide	Bethesda panel
D17S250	Dinucleotide	Bethesda panel
NR-21	Mononucleotide	Pentaplex panel
NR-24	Mononucleotide	Pentaplex panel
NR-27	Mononucleotide	Pentaplex panel
CAT-25	Mononucleotide	Additional marker
NR-22	Mononucleotide	Additional marker

**Table 5:** Performance of the NGS-based evaluation method compared to PCR and IHC.

Comparison	Sensitivity (%)	Specificity (%)	Concordance (%)
NGS vs. PCR	100	100	100
NGS vs. IHC	100	82.35	90.32

**Table 6:** Mean values of total mutations within microsatellite regions in relation to number of reads (%).

Gen	Mean Value of Total Mutations (%)
*TMPRSS2*	578.40
*SLC7AB*	333.05
*KIT*	318.80
*BCL2L11*	228.33
*ETV1*	223.25
*TET2*	203.42
*STT3A*	201.36
*ROS1*	184.56
*CASC11*	183.77
*BRAF*	179.84

**Figure A1:**
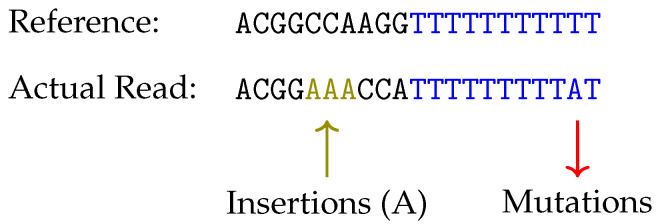
Testcase 1.

**Figure A2:**
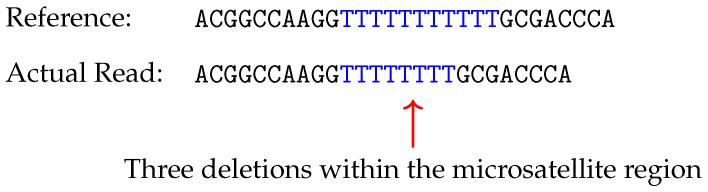
Testcase 2.

**Figure A3:**
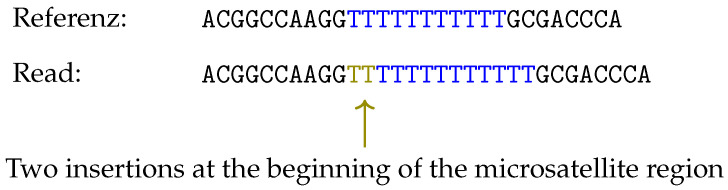
Testcase 3a.

**Figure A4:**
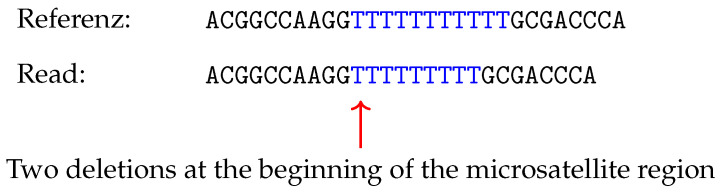
Testcase 3b.

## Data Availability

The original contributions presented in this study are included in the article/Supplementary Materials. Further inquiries can be directed to the corresponding author.

## Supplementary Materials

The following supporting information can be downloaded at: https://www.mdpi.com/article/10.3390/cancers18030433/s1, Table S1: Panel composition; Table S2: NGS evaluation per patient; Table S3: Statistic analysis of genes.

## Abbreviations

The following abbreviations are used in this manuscript:

BAM	Binary Alignment/Map
dMMR	Deficient mismatch repair
ICI	Immune checkpoint inhibitors
IHC	Immunohistochemistry
FFPE	Formalin-fixed, paraffin-embedded
MAPQ	Mapping quality
MMR	Mismatch repair
MSI	Microsatellite instability
MSI-H	High microsatellite instability
MSI-L	Low microsatellite instability
MSS	Microsatellite stability
NGS	Next-generation sequencing
PCR	Polymerase chain reaction
pMMR	Proficient mismatch repair
ROC	Receiver Operating Characteristic
SAM	Sequence Alignment/Map
TMB	Tumor mutational burden

## Appendix A. Test Cases

To validate the functional correctness of the Python-based analysis workflow, a series of self-designed test cases was generated. These tests aimed to evaluate the script’s ability to detect insertions, deletions, and base substitutions within microsatellite regions.

The test scenarios were constructed to reflect common sources of analytical error. Collectively, they assessed whether the script correctly assigned mutations to the microsatellite region and counted them individually where appropriate.

The first test case investigated whether insertions or deletions located upstream of a microsatellite could shift the apparent microsatellite position within the read, thereby hindering the detection of genuine alterations within the microsatellite region. Despite the presence of three upstream insertions, the script correctly identified a single mutation at the terminal position of an 11-T microsatellite, demonstrating that upstream indels do not impair mutation attribution within the microsatellite region.

The second test case focused on multiple indels occurring within the microsatellite region itself. Such events frequently contribute to microsatellite length variability and require precise detection. Using an example containing three deletions within the repeat tract, the script successfully recognized each deletion as an individual mutational event.

The third test case examined indels occurring at the boundary of the microsatellite. Two sub-scenarios were considered: insertions at the beginning of the microsatellite and deletions at the same position. In both instances, the script accurately classified the indels as microsatellite-associated events and reported the correct number of mutations.
